# Explaining Differential Reporting of Victimization between Parents and Children: A Consideration of Social Biases

**DOI:** 10.3390/bs3030473

**Published:** 2013-08-16

**Authors:** Sufna Gheyara John, Lisabeth F. DiLalla

**Affiliations:** 1 Department of Psychology, Southern Illinois University, MC 6502, Carbondale, IL 62901, USA; E-Mail: s.gheyara@siu.edu; 2 Department of Family and Community Medicine, Southern Illinois University School of Medicine, MC 6503, Carbondale, IL 62901, USA

**Keywords:** peer victimization, emotional intelligence, cognitive bias, emotional bias, differential reporting

## Abstract

Studies have shown that children and parents provide different reports of children’s victimization, with children often reporting more victimization. However, the reason for this differential reporting is unclear. This study explored two types of social biases (emotion recognition and perceived impairment) in parents and children as possible reasons underlying differential reporting. Six- to 10-year-old children and one of their parents were tested in a lab. Testing included subjective measures of parent alexithymic traits, child perceived impairment from victimization, and child- and parent-reported frequency of children’s peer victimization and internalizing and externalizing difficulties. Parents and children also completed an objective measure of emotion recognition. Both types of social bias significantly predicted reports of children’s peer victimization frequency as well as internalizing and externalizing difficulties, as rated by parents and children. Moreover, child perceived impairment bias, rather than parent emotion bias, best predicted differential reporting of peer victimization. Finally, a significant interaction demonstrated that the influence of child perceived impairment bias on differential reporting was most salient in the presence of parent emotion bias. This underscores the importance of expanding interventions for victimized youth to include the restructuring of social biases.

## 1. Introduction

The human experience is characterized by affiliative tendencies, with evolutionary theory suggesting that our desire to socially relate to other humans serves an important adaptive function [1]. Therefore, examining the development of affiliative, or social, skills can be an important tool for understanding human relations. Many factors contribute to the ways in which we relate to others, most of which fall under the umbrella term of “emotional intelligence” [2]. Levels of emotional intelligence vary among individuals and have been associated with negative social outcomes, such as victimization [3] and both internalizing and externalizing problem behaviors [4], all of which can have dramatic impacts on mental health and general quality of life. Therefore, a better understanding of how emotional intelligence relates to victimization can be critical for informing intervention programs. 

In studying peer victimization, researchers have gathered information from parents, teachers, peers, and the victims themselves. However, noticeable differences between victimization reports emerge based on informant identity, such that the inter-rater reliability between victims and these other individuals is poor [5,6]. Numerous studies suggest that victims tend to report more victimization than their parents [7,8]. However, the reason for this differential reporting is unclear. Examination of emotional intelligence may provide some insights into the ways in which people relate to others and interpret others’ behaviors.

### 1.1. Emotion Recognition

One core component of emotional intelligence is emotion recognition (ER), which refers to the ability to accurately recognize the feelings of those around us [9,10]. We interpret the feelings of others from both verbal (what they’re saying) and non-verbal (how they’re saying it) cues. One of the most important non-verbal cues of emotion is facial expressions because they convey salient affective information. Moreover, facial emotion recognition is a skill that develops as early as a few months of age [11] and holds particular salience for children [12,13]. Therefore, facial emotion recognition skills, as opposed to other non-verbal social cues, were assessed in this study. 

Examination of ER biases (having a bias or tendency to interpret others’ emotional cues as one particular emotion, such as fear, especially in situations of ambiguity), rather than simple ER accuracy, provides a more comprehensive view of ER abilities. One meta-analytic review identified negative emotion biases (the tendency to report that a face depicts more negativity than it does) in many individuals with mental health concerns and social difficulties, including those with unipolar and bipolar depression and a variety of anxiety disorders [14]. A particular bias toward fearful faces also has been demonstrated in spatial attention tasks with infants, younger children, and adults [15]. A second meta-analytic review concluded that fearful/threat-related attention biases have been demonstrated repeatedly in those who are anxious [16], which represents a high-risk group for victimization [17,18]. Therefore, fearful emotion biases were chosen as a focus for this study, although it is important to note that other emotion biases could also impact reporting. In addition to emotion recognition biases, it also is important to examine more sophisticated emotional difficulties in adults. Such difficulties are captured by the trait of alexithymia, or a deficiency in understanding, describing, or processing emotions [19]. These emotional skills/traits could have an impact on the ability to recognize and report victimization and psychological symptoms in oneself and in one’s children.

### 1.2. Victimization

Peer victimization is a frequent and debilitating problem faced by many school-aged children. As many as 50% of children in the United States experience at least one form of victimization [20]. In a cross-national study of 11- to 15-year-old students from 25 countries, involvement in bully-victim relationships varied from 9% to 54% [21]. Moreover, prevalence rates have been found to be around 20% for children as young as six years old [22]. Victimization has been categorized in a number of ways. One of the most common divides victimization into two distinct types, overt and relational victimization. Overt victimization is characterized by physical and verbal assault [23]. Relational victimization, on the other hand, uses relationships to inflict social harm [24]. The present study examined both types of victimization.

### 1.3. The Relation between Emotion Recognition and Victimization

How we interpret the feelings of others influences our behaviors towards them. According to social information processing theory [25], the way we encode and interpret social cues (such as facial expressions of others) informs the behavioral responses we select. Lower ER abilities in children have been linked to less sophisticated social problem solving skills [26]. In victims, the interpretation of social cues is often skewed, and as a result, behavioral reactions are often limited or inappropriate. Minor discrepancies that may have amounted to nothing can end up as large confrontations [27]. 

ER biases may be more important for influencing social behavior than the actual victimizing event [22]. Additionally, repeated victimization may contribute to the development of attribution biases and other cognitive distortions that further perpetuate this cycle. In this way, children’s ER biases can directly influence the perception, and subsequent reporting, of victimization. Similarly, parental alexithymic traits likely impact how parents report victimization experiences in their children. Parents demonstrating alexithymic traits tend to be less accurate in gauging their children’s emotional expressions and estimating the emotional abilities of their children [19]. The first goal of the present study was to examine the influence of ER biases and alexithymic traits on the reporting of social problems by both parents and children. 

### 1.4. Psychological Outcomes Associated with Peer Victimization

Experiencing victimization has been associated with a variety of negative outcomes, including loneliness, emotional dysregulation, internalizing and externalizing problems, and low self-worth [28,29,30,31,32]. Relational victimization, in particular, is related to higher levels of depression, loneliness, and lower self-worth in adolescents of both genders [33,34], although the effects are stronger in females [33]. Victims also may engage in aggressive behaviors and other externalizing problems [32,35]. Such psychopathological behavior was shown to be a consequence, rather than a cause, of the victimization experience in a sample of seventh and eighth grade students [30]. Both internalizing and externalizing symptoms may lead to a pattern of maladaptive psychosocial functioning that contributes to the repetitive nature of victimization. In this way, victims may be caught in a cycle that intensifies with time [36]. Thus, victimization is related to several psychological symptoms that can impact the quality of life for youth. The second goal of this study was to explore the psychological outcomes of victimization experiences and the impact of social biases on differential reporting of these outcomes.

Individual social biases may impact reporting style. One way this bias can develop is through distortions in the way an individual encodes emotional information from his or her environment (the emotional component of social bias examined in this study). A second path to individual social bias is that more impairing events may be more salient to a child and, thus, their frequency may be over-reported. If no perceptual bias were present, the impact of an event (in this case perceived impairment) would not influence the report of its frequency. However, victimization research supports the presence of a perceptual social bias in victimized populations that may result in over-reporting victimization frequency. For example, one study demonstrated that adolescents who reported psychosocial difficulties as a result of victimization (*i.e*., who perceived impairment from those experiences) were more likely to perceive neutral peer behavior as aggressive, and therefore identified themselves as victims more often [32]. 

### 1.5. Current Study

The first goal of this study was to explore two types of *social biases* as possible explanations for the differential reporting of victimization frequency and psychological symptoms between parents and children. *Emotion recognition bias* was defined as a bias in emotion identification, and *perceived impairment bias* was defined as a bias in the way individuals perceive the outcome of an event. First, we hypothesized that parents would report less frequent victimization than their children. Second, we hypothesized that social biases, specifically difficulty in correctly recognizing fear in oneself and others (emotion recognition bias) and interpretation of the effects of being victimized (perceived impairment bias), would be related to an individual’s reporting of both victimization and psychological symptoms. Finally, we hypothesized that both types of social biases would be related to differential reporting of victimization frequency and psychological symptoms. We also hypothesized that child and parent biases would interact such that child and parent reports of victimization would be very similar if neither reporter showed social biases, but the difference between their reports would be multiplicatively larger if both parents and children showed social biases. The second goal of this study was to examine the relation between victimization and psychological symptoms (as reported by parents and children). We hypothesized that victimization frequency, as well as social biases, would predict parent and child reported measures of internalizing and externalizing difficulties.

## 2. Method

### 2.1. Participants

Participants in this study were 58 families who came to a university lab at a rural, Midwestern university. All families had twins, triplets, or quadruplets, but for this study one child was randomly chosen from each family in order to examine the relations between parent and child behaviors. One child was missing facial bias data because of a computer malfunction, so the total number of families for analyses was 57. Children were between the ages of six and 10 years (mean age = 8.55 years, sd = 1.58), and 50% were male. Parents reported that 84% were Caucasian, 7% were African American, and 9% were of mixed race. Socioeconomic status was based on self-reported mothers’ and fathers’ education and total family income. Education for both parents ranged from high school degree only (25% for mothers, 39% for fathers) to having an advanced degree (14% for mothers, 16% for fathers), with the median for both mothers and fathers/partners being a college degree. Total income ranged from earning less than $5,000/year (2%) to earning more than $90,000/year (40%), with a median income of $85,000–$90,000/year. Most families already were part of a preschool longitudinal twin study, the Southern Illinois Twins and Siblings Study [37], and were recruited to participate in the current study. Five families were newly recruited from posting local advertisements and from asking local teachers and daycare workers if they would pass recruitment letters to families of twins.

### 2.2. Procedures

Families came to a university lab for approximately one hour to complete a battery of tests. After signing consent (parent) and assent (child) forms, each child was taken into a separate room with a trained tester and the parent was seated at a table in the waiting room with questionnaires to complete. The parent was also accompanied by a research assistant. Mid-way through the questionnaires, the parent was led by the research assistant to a computer to complete the facial expressions task and then was led back to the table to complete the questionnaires. 

Meanwhile, children were interviewed separately in private rooms, with the doors slightly ajar for safety and with music playing in the background so that they could neither hear other voices nor be heard by others in the lab. As part of the test session, the tester administered measures of internalizing and externalizing difficulties as well as a victimization and bullying questionnaire. Each child was allowed to answer questionnaires privately by listening to the tester read each item aloud, then circling the correct answer for each item on an answer sheet. The child pulled a cover sheet down after each answer so that the tester could not see the responses, although the tester could see the next item number to ensure that the child was on the correct line. Children were told that their answers were completely confidential and would not be told to their parents or anyone else. Mid-way through the testing session, children turned to a computer in the room and completed the emotion faces task (described below).

When testing was completed, children were brought back to the waiting area and were allowed to choose a toy to take home. Families were reimbursed $50 for participating.

### 2.3. Measures

#### 2.3.1. Family Demographic Information

Socioeconomic status information was collected from a questionnaire completed by the parents. This questionnaire asked about family income, education and occupation levels of both parents, race of children, and number of family members living in the home.

#### 2.3.2. Victimization and Bullying

An adapted version of Mynard and Joseph’s Multidimensional Peer-Victimization Scale (MPVS) was created that included the original victimization items plus additional items that were modified from the original items to reflect victimizing others (bullying) instead of being victimized by others [38,39]. For example, a specific victimization item (e.g., other kids have kicked me) was reworded to reflect a bullying behavior (e.g., I have kicked other kids). The altered version is called the Multidimensional Peer Victimization and Bullying Scale (MPBVS). Neutral questions were added to this measure to ensure that all items would not focus solely on negative behaviors and to make the purpose of the measure less obvious to the participants. Participants answered using a 5-point, Likert-type scale. To distinguish bullying from general aggression, the behaviors that were reported on this measure were repeated over time and included intent to inflict harm. For the present study, only victimization items were used.

Parents and children completed the same questionnaire, although the parent form was reworded to clarify that the items were about “my child.” The original four victimization scales included verbal victimization, social manipulation victimization, attacks on property victimization, and physical victimization. For the present sample, internal reliabilities for the parent victimization scales were good, with Cronbach’s alphas of 0.76, 0.82, 0.85, and 0.91, respectively. For the children, internal reliabilities for victimization scales were acceptable, with Cronbach’s alphas of 0.76, 0.69, 81, and 0.78, respectively. The physical and attacks on property victimization scales were combined to create the overt victimization score (Cronbach’s alpha = 0.82 for children and 0.90 for parents), whereas the social manipulation and verbal victimization scales were combined to create the relational victimization score (Cronbach’s alpha = 0.85 for children and 0.88 for parents). 

Children also were asked about the degree to which relational and overt victimization impaired them at home, at school, and in general. Three items for each of the two types of victimization asked “If these happened, how much did it cause you to have problems at school?”, “Have much did these cause you to have problems at home?”, and “How much have these experiences made you stay away from doing things that you would like to do?” [40]. Internal reliabilities for the three items were moderate, alpha = 0.56 for overt victimization and 0.69 for relational victimization.

#### 2.3.3. Emotion Recognition Skills

An objective measure of emotion recognition, the Diagnostic Analysis of Nonverbal Accuracy (DANVA2) [41], was administered on the computer. Children and their parents viewed 24 pictures of children’s emotional faces (12 male, 12 female) presented on the computer screen for two seconds each and identified which of four emotions was being expressed: happy, angry, sad, or fearful. The DANVA has adequate reliability. A study of similarly aged 9- to 11-year-olds showed internal consistency of 0.61 [42] and construct validity has been demonstrated in over 200 studies [41].

#### 2.3.4. Alexithymia

Parents completed the Toronto Alexithymia Scale (TAS-20), a 20-item measure designed to assess troubles identifying and describing emotions, as well as the tendency to minimize emotional experience and focus more on external stimuli [43]. The scale yields three subscales, including Difficulty Describing Feelings, Difficulty Identifying Feelings, and Externally-Oriented Thinking. The scale had moderate internal consistency for the first two subscales in our sample, Cronbach’s alpha = 0.70 and 0.73, respectively, and poor reliability for Externally-Oriented Thinking, 0.36, which we therefore did not include in our analyses. Good test-retest reliability has been shown (0.77, *p* < 0.01) [43]. Moreover, the three subscales demonstrate congruent validity and have been found to be stable and replicable in both clinical and nonclinical samples [43,44].

#### 2.3.5. Child Internalizing and Externalizing Problems

This study utilized both parent- and child-report measures to examine childhood externalizing and internalizing problems. The Child Behavior Checklist (CBCL), a 113-item measure designed to assess child behaviors and emotions during the previous six months [45], was utilized as the parent-report measure of internalizing and externalizing difficulties. In this measure, parents are asked to report how accurately an item describes their child, with answer choices of 0 (never true), 1 (sometimes true), and 2 (often true). The CBCL questionnaire consists of eight empirically-based subscales, including anxious/depressed, withdrawn/depressed, somatic complaints, social problems, thought problems, attention problems, rule-breaking behavior, and aggressive behavior. The anxious/depressed, withdrawn/depressed, and somatic complains subscales form the Internalizing Problems composite and the aggressive behavior and rule-breaking behavior subscales form the Externalizing Problems composite, both of which were used in analyses for this study (Cronbach’s alpha = 0.83 for Internalizing and 0.91 for Externalizing). Because this sample was non-clinical, raw scores rather than T scores were used for analyses.

Children completed the Strengths and Difficulties Questionnaire (SDQ), a 25-item measure assessing behavioral and emotional symptoms in children ages 3–16 years [46]. We adapted the parent version for use with the children in this study, using pictorial aids for children to look at when choosing their answers. Children were given a prompt and then asked to respond with how accurately it describes them (0 = not true, 1 = somewhat true, and 2 = certainly true). A higher score on the maladaptive subscales reflects greater difficulty. Items form four maladaptive subscales: emotional symptoms, conduct problems, hyperactivity/ inattention problems, and peer problems (and a prosocial behaviors scale which was not used in this study). Internal consistency reliability was moderate for this sample: 0.66, 0.52, and 0.58, and 0.49, respectively. Two overall scales were used that have been found to be more reliable in non-clinical samples such as the present one [47]: Externalizing (conduct problems plus hyperactivity/inattention problems) and Internalizing (emotional symptoms plus peer problems). These two higher-order scales showed adequate reliability within our sample, Cronbach’s alpha = 0.72 and 0.74, respectively. These two scales also were preferred because they more closely parallel the CBCL super-factors. The child version of the CBCL could not be used with this sample because it is only normed down to age 11 years and is much too long for use with this young age group. However, the constructs are very similar across these two measures. For both, Internalizing captures anxiety, depression, and somatic problems, and Externalizing captures aspects of acting out against others, breaking rules, and difficulty paying attention. The SDQ has been shown to be comparable to the CBCL in terms of parent ratings of internalizing and externalizing [48] but its much shorter length made it more amenable to testing for the young children in this sample.

## 3. Results

Prior to analyses, variables were examined for skewness and corrected accordingly. Means and standard deviations prior to transformation for all study variables are included in Table 1. As can be seen, victimization scores ranged from 0 to 27 for parent raters and from 0 to 25 for child raters. For children’s ratings, 25% of children scored 0–2, 50% scored 3–10 for overt and 3–12 for relational, and 25% scored 11–25 for overt and 13–24 for relational. Parents showed less variability. For parents’ ratings of overt victimization, 50% of children received a score of 0 and only 25% scored 3 or higher. For relational victimization, 24% received a score of 0 and 50% received a score of 3–27. Thus, in general, parents rated children as receiving less victimization than children themselves reported. Additionally, child reports of victimization and impairment were significantly correlated (see Table 2), although this correlation was certainly not unity, demonstrating that these measures were assessing different experiences. About one third of children reported no impairment (score of 3) from being victimized (40% for relational, 31% for overt). For relational victimization, about 30% scored between 4 and 8, and 20% scored between 9 and 15 on the impairment scale. For overt victimization, 33% scored between 4 and 6 and 36% scored between 7 and 15. Thus, as with victimization, there was a wide range of perceived impairment from victimization in this non-clinical sample.

**Table 1:** Descriptive Statistics for All Study Variables Prior to Transformation.

		Actual Range		Possible Range	
	Untransformed Mean (s.d.)	Min	Max	Min	Max
**Parent Measures**					
DANVA Parent Fearful Bias ^a^	0.57 (0.99)	0	5	0	18
Parent TAS Difficulty Describing Feelings ^b^	9.36 (3.72)	5	21	5	25
Parent TAS Difficulty Identifying Feelings ^b^	11.07 (3.92)	7	22	7	35
Parent-reported Relational Victimization ^b^	4.22 (5.07)	0	27	0	28
Parent-reported Overt Victimization ^a^	2.47 (4.29)	0	24	0	28
CBCL Internalizing ^b^	5.43 (5.60)	0	27	0	62
CBCL Externalizing ^b^	9.09 (8.42)	0	37	0	66
**Child Measures**					
DANVA Child Fearful Bias ^b^	0.86 (1.03)	0	4	0	18
Child Relational Impairment	6.02 (3.30)	3	15	3	15
Child Overt Impairment ^b^	5.81 (2.91)	3	15	3	15
Child-reported Relational Victimization ^b^	7.52 (6.32)	0	24	0	28
Child-reported Overt Victimization ^b^	7.52 (6.56)	0	25	0	28
SDQ Internalizing	16.41 (4.34)	10	30	10	30
SDQ Externalizing	16.50 (3.87)	10	25	10	30
Child Minus Parent Overt Victimization	5.05 (6.57)	−12	23	−28	28
Child Minus Parent Relational Victimization	3.29 (6.33)	−13	16	−28	28

^a^ Cube rooted to correct for skewness; ^b^ Square rooted to correct for skewness.

**Table 2:** Intercorrelations among Study Variables.

	C-FB	P-FB	TASIF	TASRF	P-OV	P-RV	C-OV	C-RV	C-OI	C-RI	CBCLExt	CBCLInt	SDQExt
C-FB	1.0												
P-FB	0.10	1.0											
TASIF	−0.23	0.02	1.0										
TASRF	−0.26+	−0.02	0.59 **	1.0									
P-OV	−0.05	−0.33+	0.08	0.21	1.0								
P-RV	−0.01	−0.11	0.30+	0.32+	0.60 **	1.0							
C-OV	0.05	−0.17	−0.09	−0.12	0.29+	0.24	1.0						
C-RV	0.24	−0.09	−0.11	−0.17	0.28+	0.42 **	0.72 **	1.0					
C-OI	0.43 **	0.02	−0.09	−0.06	0.04	0.10	0.41 **	0.59 **	1.0				
C-RI	0.31+	−0.13	−0.09	−0.05	0.07	0.08	0.43 **	0.55 **	0.81 **	1.0			
CBCLExt	0.16	−0.08	0.23	0.14	0.36 *	0.31+	0.00	0.04	0.04	0.00	1.0		
CBCLInt	−0.04	−0.10	0.37 *	0.32+	0.31+	0.33+	−0.17	−0.20	−0.25	−0.31+	0.61 **	1.0	
SDQExt	0.20	0.00	−0.18	−0.12	0.10	.014	0.39 *	0.49 **	0.33 *	0.16	0.14	−0.06	1.0
SDQInt	0.29+	−0.15	−0.05	−0.12	0.23	0.23	0.43 **	0.51 **	0.48 **	0.41 *	0.30+	0.12	0.47 **

Note: C-FB = Child Fearful Bias; P-FB = Parent Fearful Bias; TASIF = parent TAS Difficulty Identifying Feelings; TASRF = parent TAS Difficulty Reporting Feelings; P-OV = Parent-report Overt Victimization; P-RV = Parent-report Relational Victimization; C-OV = Child-report Overt Victimization; C-RV = Child-report Relational Victimization; C-OI = Child Overt Impairment; C-RI = Child Relational Impairment; CBCLExt = CBCL Externalizing; CBCLInt = CBCL Internalizing; SDQExt = SDQ Externalizing; SDQInt = SDQ Internalizing; + *p* < 0.05; * *p* < 0.01; ** *p* < 0.001.

### 3.1. ER Biases and Alexithymia as They Relate to Reports of Victimization and Impairment

Pearson correlations were calculated between child and parent ER biases of fear (reporting faces as showing fear when they actually were showing another emotion) and parent alexithymia (difficulty identifying feelings and difficulty describing their own feelings). These also were correlated with parent and child reports of overt and relational victimization, child reports of perceived impairment from victimization, and parent and child reports of internalizing and externalizing difficulties. These correlations are presented in Table 2. Because of the large number of correlations, a more stringent cut-off of *p* < 0.01 was used to determine whether there was a significant correlation between two variables.

#### 3.1.1. Child *versus* Parent Reports of Victimization

We first tested whether parents reported less victimization than children. Two paired-sample t-tests showed that children reported significantly more overt (*t*(57) = 9.03, *p* < 0.001) and relational (*t*(57) = 4.32, *p* < 0.001) victimization than did their parents, which supported our first hypothesis.

#### 3.1.2. Social Biases

Our second hypothesis was that both forms of social bias would be related to reporting of both victimization and psychological symptoms. As can be seen from Table 2, children’s fear biases as assessed on the DANVA were significantly correlated with their own reports of impairment from victimization, but not with frequency of victimization. Parents’ DANVA fear biases were only marginally related to their own reports of frequency of child overt victimization. However, parent alexithymia was marginally correlated with parent reports of relational victimization, and was significantly correlated with CBCL Internalizing, such that parents with greater difficulty identifying and describing feelings also rated their children as having more internalizing problem behaviors. In addition, child perceived impairment was significantly correlated with child reports of both overt and relational victimization but not with parent reports of victimization. Finally, child reports of victimization and impairment were significantly correlated with child-reported SDQ internalizing and externalizing problems. Thus, our second hypothesis was partially supported. Although correct recognition of fear faces was not significantly correlated with reports of victimization, children with a greater fear bias were more likely to report greater impairment from victimization. In addition, parents with an emotion recognition bias (poor TAS scores) were more likely to rate their children as having internalizing problem behaviors. 

#### 3.1.3. Social Biases and Differential Reporting

Finally, regression analyses were used to test the hypothesis that both types of social biases would be related to differential reporting of victimization frequency and psychological symptoms and that there would be an interaction between child and parent biases. The dependent variables were difference scores between child and parent reports of victimization created separately for overt and relational victimization by subtracting parent scores from child scores for each type of victimization. We only included TAS difficulty identifying feelings (DIF) in the regression equations because the two TAS scales were highly inter-correlated, they showed similar patterns of correlations across the other variables, and only DIF reached a conservative level of significance. We included age in the model first to partial out its effect. Then we entered child fearful bias, child perceived impairment, and parent DIF, all centered, in step 2. Finally, we entered the interaction terms in step 3 (see Table 3).

**Table 3:** Hierarchical Multiple Regression Analyses Predicting Differences in Reports of Victimization (Child Minus Parent Scores).

		Difference in Overt Victimization		Difference in Relational Victimization	
		∆R^2^	β	∆R^2^	β
**Step 1**		0.10 *			
	Age		−0.32 *		−0.41 **
**Step 2**		0.10		0.25 ***	
	Child Fearful Bias Centered		−0.04		0.16	
	Parent TAS DIF Centered		−0.06		−0.22
	Perceived Impairment Centered		0.35 *		0.41 **
**Step 3**		0.08		0.01	
	TASDIF X Impairment		0.34 *		−0.09	
	TASDIF X Child Fearful Bias		−0.11		0.11

Note: For overt victimization, *F*(6,56) = 3.36, *p* = 0.007, adjusted *R*^2^ = 0.20. For relational victimization, *F*(6,56) = 6.27, *p* < 0.001, adjusted *R*^2^ = 0.36; * *p* < 0.05; ** *p* < 0.01; *** *p* < 0.001.

**Figure 1:**
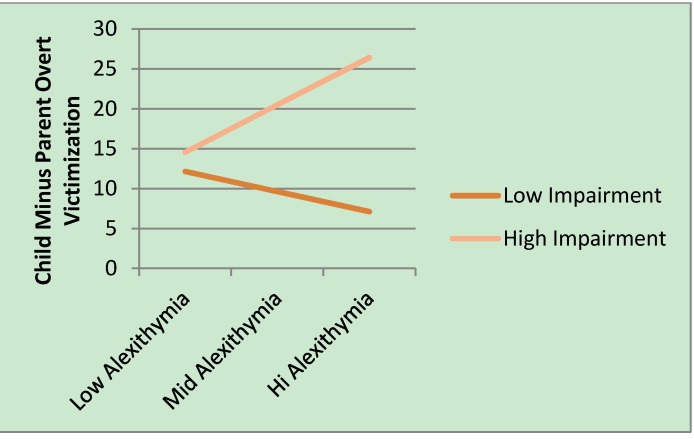
Interaction between TAS difficulty identifying feelings and child reports of perceived impairment from overt victimization as they predict differences in parents’ and children’s overt victimization reports.

Both full models were significant. Results showed that after accounting for age, child perceived impairment significantly predicted difference scores in both overt and relational victimization. In addition, the interaction between impairment and parent difficulty identifying feelings significantly predicted differences in reports of overt victimization. The interaction is plotted in Figure 1 using the simple slopes and y-intercepts. Child impairment lines were plotted separately for children above the mean (high impairment) and at or below the mean (low impairment). Alexithymia was grouped as 1 s.d. or more below the mean (low alexithymia), between 1 s.d. below and 1 s.d above the mean (mid alexithymia), and 1 s.d. or more above the mean (high alexithymia). As can be seen in the figure, differential reporting of overt victimization did not differ when parents had no alexithymia, regardless of the degree to which children reported perceived impairment. However, when parents had difficulty identifying feelings, the difference between parent and child reports of overt victimization significantly increased as children’s degree of impairment increased. Specifically, when levels of parent alexithymia were moderate or high and level of impairment was highest, children reported much more victimization than their parents did. However, when alexithymia was moderate or high but level of impairment was very low, the difference between children and parents was significantly decreased.

### 3.2. Predicting Internalizing and Externalizing Problem Behaviors

Regression analyses were utilized to test the hypothesis that parent- and child-reported internalizing and externalizing problem behaviors were significantly predicted from social biases and victimization. Because of the high inter-correlations between overt and relational victimization, we ran separate regression equations for each one. 

The four dependent variables were SDQ Externalizing, SDQ Internalizing, CBCL Externalizing, and CBCL Internalizing. For all regression equations, we entered age of child in step 1, emotional recognition biases (DANVA fear for child and for parent plus TAS DIF) in step 2, child perceived impairment bias in step 3, and child and parent frequency of victimization reports in step 4. 

Results for predicting SDQ Externalizing and Internalizing scores from overt and relational victimization are presented in Table 4. The notable result is that perceived impairment from overt victimization predicted child reports of externalizing and internalizing problem behaviors and child reports of relational victimization predicted externalizing and internalizing problem behaviors. Also, child fearful bias significantly predicted child scores on internalizing problem behaviors. No parent emotion biases or parent reports of victimization predicted SDQ problem behaviors.

Similar regression analyses were repeated using parent reports of internalizing and externalizing from the CBCL (see Table 5). Overall models predicting from overt victimization were significant for internalizing as well as externalizing. The overall model predicting from relational victimization was significant for internalizing but the one for externalizing was not. 

An examination of Table 4 shows an interesting pattern quite different from child reports. Parents who had more difficulty identifying feelings (TAS) reported significantly more externalizing and internalizing problems. Also, parents who reported more victimization, both overt and relational, were significantly more likely to report more externalizing and internalizing problems. Surprisingly, parents of children who reported more perceived impairment from relational victimization were less likely to report internalizing problems. There were no significant predictions from fearful biases (parent or child) or from child reports of victimization.

**Table 4:** Hierarchical Multiple Regression Analyses Predicting Strengths and Difficulties Questionnaire (SDQ) From Emotion Biases and Victimization.

		Predicting from Overt Victimization				Predicting from Relational Victimization			
		SDQ Externalizing		SDQ Internalizing		SDQ Externalizing		SDQ Internalizing	
		∆R^2^	β	∆R^2^	β	∆R^2^	β	∆R^2^	β
**Step 1**		0.05		0.16 **		0.05		0.16 **	
	Age		−0.22		−0.40 **		−0.22		−0.40 **
**Step 2**		0.06		0.07		0.06		0.07	
	Child Fearful Bias		0.13		0.27 *		0.13		0.27 *
	Parent Fearful Bias		0.10		−0.04		0.10		−0.04
	Parent TAS DIF		−0.16		0.08		−0.16		0.08
**Step 3**		0.09 *		0.11 **		0.01		0.04	
	Perceived Impairment		0.35 *		0.38 **		0.11		0.24
**Step 4**		0.03		0.06		0.18 **		0.10 *	
	Child-reported Victimization		0.20		0.18		0.55 **		0.33 *
	Parent-reported Victimization		0.03		0.16		−0.05		0.09

Note: TAS DIF = Parent Alexithymia Difficulty Identifying Feelings. For overt victimization: SDQ Externalizing, overall model *F*(7,56) = 2.15, *p* = 0.055; SDQ Internalizing, overall model *F*(7,56) = 4.40, *p* = 0.001. For relational victimization: SDQ Externalizing, overall model *F*(7,56) = 2.91, *p* = 0.013; SDQ Internalizing, overall model *F*(7,56) = 4.00, *p* = 0.002; * *p* < 0.05; ** *p* < 0.01; *** *p* < 0.001.

**Table 5:** Hierarchical Multiple Regression Analyses Predicting Child Behavior Checklist (CBCL) From Emotion Biases and Victimization.

		Predicting from Overt Victimization				Predicting from Relational Victimization			
		CBCL Externalizing		CBCL Internalizing		CBCL Externalizing		CBCL Internalizing	
		∆R^2^	β	∆R^2^	β	∆R^2^	β	∆R^2^	β
**Step 1**		0.02		0.03		0.02		0.03	
	Age		−0.12		0.18		−0.12		0.18
**Step 2**		0.11		0.13		0.11		0.13	
	Child Fearful Bias		0.21		0.07		0.21		0.07
	Parent Fearful Bias		−0.02		−0.10		−0.02		−0.10
	Parent TAS DIF		0.32 *		0.36 **		0.32 *		0.36 **
**Step 3**		0.00		0.04		0.01		0.10 *	
	Perceived Impairment		0.02		−0.22		−0.14		−0.37 *
**Step 4**		0.13 *		0.10 *		0.08		0.09	
	Child-reported Victimization		−0.09		−0.10		−0.01		−0.12
	Parent-reported Victimization		0.40 **		0.34 *		0.31*		0.35 *

Note: TAS DIF = Parent Alexithymia Difficulty Identifying Feelings. For overt victimization: CBCL Externalizing, *F*(7,56) = 2.46, *p* = 0.030, adjusted *R*^2^ = 0.15; CBCL Internalizing, *F*(7,56) = 2.90, *p* = 0.013, adjusted *R*^2^ = 0.19. For relational victimization: CBCL Externalizing, *F*(7,56) = 2.02, *p* = 0.071, adjusted *R*^2^ = 0.11; CBCL Internalizing, *F*(7,56) = 3.60, *p* = 0.003, adjusted *R*^2^ = 0.25; * *p* < 0.05; ** *p* < 0.01; *** *p* < 0.001.

## 4. Discussion

Several studies have shown that parents and children differentially report peer victimization, with most research suggesting that parents tend to report less victimization than their children do [5]. This paper explored potential influences on this differential reporting, specifically examining two forms of social bias. Additionally, we attempted to establish clear trends in the influence of such biases on reporting in general by examining the relation of social biases to both child and parent reports of child difficulties (frequency of victimization, internalizing, and externalizing problem behaviors). We found, in agreement with past studies, that parents did indeed report less victimization than did their children. Interestingly, our results suggest that individual social biases are significantly related to the ways in which children and parents report victimization and behavioral and emotional difficulties. Therefore, when comparing the information gathered from multiple sources, researchers and clinicians alike must bear in mind the influence of such biases on the differences they observe.

Specifically, an examination of the relation between emotional biases and reports of victimization suggested that child fear biases and parent alexithymic traits of difficulties identifying and describing feelings are related to the frequency of child victimization each informant reports. Moreover, child perceived impairment bias was significantly related to child victimization frequency reports, but not parent reports. Taken together, these results indicate that children’s social biases are related to the ways in which they report victimization. Similarly, parents’ alexithymic traits (a more sophisticated form of emotional bias) are related to their reports on the frequency of their children’s victimization. 

Interestingly, the impact of social biases extends beyond frequency of victimization. When examining child-reported internalizing and externalizing difficulties, results indicated that child perceived impairment bias best predicted internalizing problems, whereas frequency of child-reported relational victimization best predicted externalizing problems. This difference indicates a differential impact of social bias related to overt and relational victimization and their impact on child-reported emotional/behavioral difficulties, with this perceived impairment bias playing a more important role in internalizing difficulties. Parent-reported measures of victimization and emotional biases were not related to child reports of internalizing or externalizing behaviors, suggesting that parent biases or reports of victimization frequency do not predict difficulties reported by children. This strengthens the notion that individual biases impact children’s own reports of difficulties. 

Examination of parent-reported internalizing and externalizing difficulties in their children suggested a different pattern, with parent emotional bias (difficulty identifying feelings, not fearful bias) predicting both internalizing and externalizing difficulties. Moreover, parent reports of overt victimization were significantly related to both types of problem behaviors, whereas relational victimization was only related to internalizing, but not externalizing, difficulties. Therefore, parents’ emotional biases predicted their perception of child difficulties, both in terms of victimization and emotional/behavioral problems. Neither child social bias significantly predicted parent reports of emotional/behavioral difficulties. Again, these results underscore a clear influence of individual bias on individual perceptions and subsequent reporting. 

Finally, we explored the impact of emotional and cognitive biases on differential reporting of victimization. In our sample, child- and parent-reported frequency of victimization was significantly correlated for both relational and victimization. However, when parent and child scores were compared, children almost always rated victimization as a more frequent occurrence than did their parents. Child perceived impairment bias best predicted differences in both relational and overt victimization reports between parents and children. Specifically, the greater impairment children perceived, the more victimization they reported compared to their parents. This suggests that children who are highly impaired by victimization may find these experiences more salient, and thus report that they happen more often than parents note. Importantly, impairment was only correlated with victimization reports about 50%, which, although significant, makes it clear that these are not measuring the same things. We also found that this perceived impairment bias differed depending on parents’ emotional biases (defined here as difficulty identifying feelings). Children reported significantly more victimization than did their parents when children viewed themselves as highly impaired by victimization and their parents had high levels of difficulty identifying feelings. However, when parents displayed an emotional bias but child perceived impairment bias was low, the difference between parent and child reported victimization significantly decreased. These results suggest that child perceived impairment bias best accounts for differential reporting of both types of victimization overall, and that the difference for overt victimization is exacerbated when parents also display an emotional bias. 

There are several strengths of this study that extend the current research literature. First, although differential reporting of victimization has been demonstrated in the literature, relatively few studies have explored the mechanisms behind these differences. This study provides a more comprehensive view of differential reporting (by including parent and child reports of each construct) that can aid in the development of assessment tools that query for such biases in addition to simple frequency counts. Indeed, emotional biases may provide a mechanism to explain reporter bias and subsequent differential reporting between informants. Second, this study utilized objective measures of ER biases, and as such, our results provide stronger evidence for the influence of emotional biases beyond simple reporting style. Finally, utilizing school-aged children (ages six to 10 years) provides a sample in which bullying and victimization is highly prevalent, and thus, results from this study could impact a large percentage of children. 

Study strengths should be considered along with limitations. First and foremost, this study has a relatively small sample size. However, power analyses through G*Power, suggest that a sample size of 43 individuals allows detection of a moderate effect size in our regression analyses [49]. Therefore, our sample of 57 individuals is capable of finding moderate effects. Second, correlations were interpreted conservatively, utilizing an alpha error probability of *p <* 0.01. Therefore, although these results should be replicated in larger samples, findings from the current study are statistically sound and interpretable. Third, our sample was fairly homogenous, with most children being of Caucasian descent and growing up in middle-to-high-income households. Generalization of these results to more diverse populations should be made with caution, and future studies should examine the role of social biases in more heterogeneous samples.

## 5. Conclusions

In summary, our results suggest that the explanation for differential reporting of victimization is a complex combination of individual social biases. It is not simply that children do not tell their parents what occurs, and thus parents are under-reporting victimization. It is much more likely that both parents’ and children’s reports of victimization are inherently influenced by their own biases. Perhaps a logical follow-up question to these results is, who is correct? That is, whose report of victimization is more valid or more accurately portrays the “real world” frequency of victimization? It is important to remember that our perceptions create the reality in which we live, and it may not matter much who is more “correct.” Rather, in helping victimized youth, we must consider that their perceptions of victimization are the reality under which they operate, and these perceptions are influenced by their own biases. 

Understandably, misinterpreting social cues can certainly lead to maladaptive social interactions and more general social skills deficits. According to social information processing theory [25], our behavioral choices are shaped by the ways in which we encode and interpret environmental cues. Moreover, previous social interactions contribute to social biases in future relations between individuals. In this way, social biases are rooted in past interactions and largely influence future relationships. Therefore, in addition to targeting social skills and problem-solving skills training for children who are bullied, cognitive work targeting these biases can help to alter children’s perceptions of their surroundings. This in turn can help to make victimization less salient and perhaps less impairing for bullied children. Additionally, given that parent biases also impact their perceptions of their children’s social experiences and difficulties, cognitive work with parents could help them understand how their own biases may be coloring the ways in which they view their children.
